# “No One Manages It; We Just Sign Them Up and Do It”: A Whole System Analysis of Access to Healthcare in One Remote Australian Community

**DOI:** 10.3390/ijerph19052939

**Published:** 2022-03-03

**Authors:** Eloise Osborn, Marida Ritha, Rona Macniven, Tim Agius, Vita Christie, Heather Finlayson, Josephine Gwynn, Kate Hunter, Robyn Martin, Rachael Moir, Donna Taylor, Susannah Tobin, Katrina Ward, Kylie Gwynne

**Affiliations:** 1 Centre for Global Indigenous Futures, Faculty of Medicine, Health and Human Sciences, Macquarie University, Sydney, NSW 2109, Australia; marida.ritha@students.mq.edu.au (M.R.); rona.macniven@mq.edu.au (R.M.); kylie.gwynne@mq.edu.au (K.G.); 2 Faculty of Medicine and Health, School of Population Health, University of New South Wales, Sydney, NSW 2052, Australia; 3 Durri Aboriginal Corporation Medical Service, Kempsey, NSW 2440, Australia; tim.agius@y7mail.com; 4 Poche Centre for Indigenous Health, University of Sydney, Sydney, NSW 2006, Australia; vita.christie@sydney.edu.au (V.C.); josephine.gwynn@sydney.edu.au (J.G.); rachael.moir@health.nsw.gov.au (R.M.); 5 Brewarrina Multipurpose Service, Brewarrina, NSW 2839, Australia; heather.finlayson@health.nsw.gov.au; 6 The George Institute for Global Health, The University of New South Wales, Sydney, NSW 2052, Australia; khunter@georgeinstitute.org.au; 7 Mid North Coast Local Health District, Coffs Harbour, NSW 2450, Australia; robyn.martin3@health.nsw.gov.au; 8 Pius X Aboriginal Medical Service, Moree, NSW 2400, Australia; ceo@piusx.com.au; 9 Honorary Faculty of Medicine and Health, University of Sydney, Sydney, NSW 2006, Australia; susannahbrodie@gmail.com; 10 Brewarrina Aboriginal Medical Service, Brewarrina, NSW 2839, Australia; katrinaw@brewarrinaams.com.au

**Keywords:** Aboriginal Australians, remote health, availability, accessibility, community-based healthcare services, healthcare services

## Abstract

Objective: To assess the accessibility, availability and utilisation of a comprehensive range of community-based healthcare services for Aboriginal people and describe contributing factors to providing effective healthcare services from the provider perspective. Setting: A remote community in New South Wales, Australia. Participants: Aboriginal and non-Aboriginal health and education professionals performing various roles in healthcare provision in the community. Design: Case study. Methodology: The study was co-designed with the community. A mixed-methods methodology was utilised. Data were gathered through structured interviews. Descriptive statistics were used to analyse the availability of 40 health services in the community, whilst quotations from the qualitative research were used to provide context for the quantitative findings. Results: Service availability was mapped for 40 primary, specialised, and allied health services. Three key themes emerged from the analysis: (1) there are instances of both underservicing and overservicing which give insight into systemic barriers to interagency cooperation; (2) nurses, community health workers, Aboriginal health workers, teachers, and administration staff have an invaluable role in healthcare and improving patient access to health services and could be better supported through further funding and opportunities for specialised training; and (3) visiting and telehealth services are critical components of the system that must be linked to existing community-led primary care services. Conclusion: The study identified factors influencing service availability, accessibility and interagency cooperation in remote healthcare services and systems that can be used to guide future service and system planning and resourcing.

## 1. Introduction

### 1.1. Health System and Services: Funding, Service Delivery and Management

Australia enjoys a comprehensive and primarily publicly funded healthcare system. This system aims to provide high-quality, cost-effective healthcare to all Australians, who tend to have high life expectancies and a low burden of disease [1]. Federal, state, and local governments share responsibilities for healthcare funding, service delivery and service management [2]. The federal government is responsible for the largest proportion of healthcare expenditure through Medicare and the Pharmaceutical Benefits Scheme (PBS), while state and territory governments are responsible for service delivery and health infrastructure [2,3]. Private companies and non-governmental organisations (NGOs) provide a smaller but significant proportion of Australia’s health services. Aboriginal Community Controlled Health Organisations (ACCHOs) are an important part of the healthcare system designed specifically for Aboriginal people, providing holistic, culturally safe, and high-quality healthcare and health education services [4,5].

### 1.2. Health Inequality: Aboriginal and Torres Strait Islander People

Aboriginal people remain resilient and continue to celebrate a strong connection with their land, culture, knowledge, and traditions. However, health inequalities and inequities exist between Aboriginal and non-Aboriginal Australians. These inequalities stem from the ongoing impacts of colonisation, including the displacement and dispossession of land and resources, the removal of children, erasure and disruption of cultural practices, customs and languages, and the ongoing intergenerational trauma and racial discrimination [6]. Aboriginal and Torres Strait Islander people experience a life expectancy gap of around 10 years, higher infant and maternal mortality rates, higher prevalence of long-term chronic illnesses, higher burden of diseases and poorer perceived health status in comparison to non-Aboriginal Australians [6,7,8].

In 2008, the Council of Australian Governments commenced the Closing the Gap strategy with a purpose to close the gap in life, education, employment and health outcomes of Aboriginal and Torres Strait Islander people within a generation [9]. The strategy included responsibilities at all levels of government and national reporting. Twelve years later and despite government effort, there has been poor progress towards many of the targets. The gap in life expectancy between Aboriginal and non-Aboriginal Australians is widening, and infant mortality is 2.4 times the rate of non-Aboriginal Australians [10]. The Closing the Gap strategy has been redesigned by the government, in negotiation with Aboriginal people and organisations, with new key performance indicators. These new targets focus on determinants of health, education, and employment, including reducing child removals and incarceration rates, as well as supporting the preservation of Aboriginal people’s connections to their land, culture and languages [11].

### 1.3. Rural and Remote Health

Australians living in small towns or communities in rural and remote Australia tend to have poorer access to healthcare and worse health outcomes than people living in higher-density urban and metropolitan areas [12]. Regional and remote areas often have limited local health services available, with fewer staff and resources, resulting in a reduced capacity to provide primary healthcare services [13]. When accessing more specialised services, people living in rural and remote areas are often required to travel to regional centres and cities or rely on less frequent fly-in–fly-out services. This reduced availability of health services is associated with higher rates of preventable hospitalisations, particularly in remote and very remote communities [14]. These disparities between remote, rural and urban healthcare access compound health inequalities and inequities between Aboriginal and non-Aboriginal Australians [15]. Aboriginal people are more likely than non-Aboriginal people to live in very remote and isolated regions, with extremely limited access to healthcare facilities [16].

### 1.4. Importance of Culturally Safe Care

Providing culturally safe, respectful and appropriate healthcare is essential to improve healthcare access and outcomes for Aboriginal people [4,5]. Mainstream health services often struggle to meet the needs of Aboriginal patients in culturally safe ways. Aboriginal patients tend to access healthcare services later in the disease or illness process, which can lead to poorer health outcomes [17]. A reason for this is that Aboriginal patients often have a mistrust of healthcare services and experience stigmatisation and discrimination [18]. Fears often stem from discriminatory government policies, particularly the removal of Aboriginal children from their families [19]. ACCHOs have been highly effective in addressing these barriers, with a focus on culturally safe care resulting in improved trust, access, understanding and health outcomes among Aboriginal patients [4,5]. Trust is foundational for beneficial relationships, particularly when people are vulnerable and fearful due to health concerns for themselves and their families [20].

### 1.5. Justification of Study

Qualitative findings have highlighted [21] the complexity and fragmentation of Australian health-service delivery between different levels and sectors of government and private organisations and NGOs. This can make the system difficult to navigate for both consumers and service providers. Poor coordination of healthcare services continues to be a barrier for providing and accessing holistic healthcare, particularly in rural and regional Australia [13]. Additionally, healthcare recipients often need to travel to regional centres or cities to access care that is not available closer to home [22]. These barriers tend to have an increased impact on the health outcomes and access to health services among Aboriginal people in comparison to other Australians living in remote areas [15]. This paper provides a detailed analysis of health service coordination issues and their impacts on patients, staff, families and communities through the lens of one remote community. We also examine how local staff manage significant and complex community needs.

## 2. Methods

Ethics approval for this study was granted by the NSW Aboriginal Health & Medical Research Council (AH&MRC) Ethics Committee (1173/16).

This study utilized a mixed methodology with a case study design and was conducted in a remote town in NSW, with a high (>65%) Aboriginal population. This remote town had two local schools and three local health services. The distance from the study town to the closest major regional city is almost 400 km away, and public transport to this city is not available. The case study is nested within a wider study conducted in 2016 [21].

This study involved a team of Aboriginal and non-Aboriginal researchers with an established rapport with each study community, working together over several years of co-designed research. Aboriginal community members and local ACCHOs worked with researchers to co-design a research protocol reflecting the concerns and priorities of the local community as well as culturally safe practice. Two non-Aboriginal female authors—a researcher (K.H.) and a project manager (R.M.)—conducted interviews. Both interviewers were experienced in conducting interviews in Aboriginal communities and had several years of experience working in Aboriginal health and ongoing cultural training and mentorship. The full protocol for the original mapping study is described elsewhere [23].

The study utilized snowball sampling to recruit Aboriginal and non-Aboriginal health and education professionals from multiple local service providers. Local school staff were included in the study as local schools act as a hub for visiting and local health services for children and their families. Data were collected through structured interviews conducted face-to-face or over the phone and were conducted in the workplace or in the community, depending on participant preference. All interviews were audio-recorded, and transcribed verbatim. Interviews focused on the availability, accessibility and frequency of primary healthcare, specialist medical care and allied health services in the community. Participants were asked about the availability of a comprehensive list of 40 health services in the community. The frequency of service availability was measured on a 9-point scale from “always” to “never” (including the responses “weekly”, “monthly” and “annually”). This scale was condensed into a 6-point scale for the case study to reflect the most used responses, with footnotes for infrequently used responses. Service delivery types were recorded under the categories of “local”, “drive-in–drive-out” (DIDO), “fly-in–fly-out” (FIFO), “telehealth” and “patient required to travel”. Participants were given opportunities to give longer open answers to questions about how services could be improved, barriers to healthcare access for Aboriginal people in the community, and how local staff managed systemic barriers to health access and participation in their community. Interviews were between 12 and 90 min in duration, with an average interview length of 35 min.

This study analysed findings from interviews with 11 participants, across 3 local health services (n = 4), 2 of whom were from an ACCHO, 2 local schools (n = 5), and 2 FIFO services (n = 2). ACCHO workers chose to be interviewed together, as did 3 of the school staff. Many of the participants identified as Aboriginal, but this was not recorded to ensure their confidentiality was protected. Participants are identified in the results and discussion by an alphabetical pseudonym to preserve their anonymity and by their occupation to contextualize their responses.

Descriptive statistics were used to analyse quantitative data. Subsequently, thematic analysis of qualitative data was used to contextualise the quantitative data with quotations and observations from the interviews to demonstrate the implications of the quantitative findings. The descriptive data analysis was used to produce two tables, demonstrating the availability of services by service delivery type and frequency of availability. The qualitative data from interviews were thematically coded and analysed using an inductive approach [24].

## 3. Results

The quantitative results of this study demonstrated the availability of primary, specialised, and allied health services by service delivery type (Table 1) and frequency (Table 2). The qualitative analysis of interviews identified three key themes to guide the interpretation of these findings:

- There are examples of both underservicing and overservicing which give insight into systemic barriers in interagency cooperation;
- Nurses, community health workers, Aboriginal health workers, teachers, and administration staff have an invaluable role in healthcare and improving patient access to health services and could be better supported through further funding and opportunities for specialised training;
- Visiting and telehealth services are critical components of the system that must be linked to existing community led primary care services.

Table 1 and Table 2 provide descriptive data, mapping the availability of 40 health services. The data indicate the services available to the community, through multiple health providers. The tables include the availabilities of all service providers used by the community. For example, there is a general practitioner (GP) who is ‘local’ and ‘always available’, as well as supplementary ‘DIDO’ and ‘FIFO’ GPs who are available ‘weekly’ and ‘fortnightly’. As this still does not always meet the patient needs, some patients are also ‘required to travel’ to access the GP.

The data on service availability in the community demonstrated that many services were available locally at varying frequencies. Primary healthcare providers including nurses, Aboriginal health and education workers, community health workers and doctors were always available locally. Frequently utilised allied health services including counsellors, dieticians and physiotherapists were available weekly to fortnightly. Highly specialised and infrequently used services including neurology and cardiology were available between one and four times a year.

### 3.1. Service Availability

**Figure 1:**
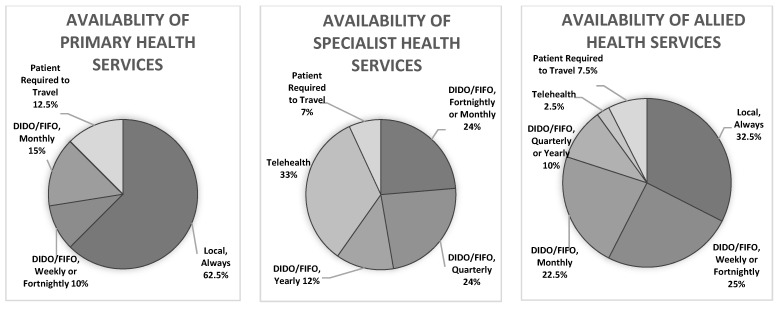
Availability of primary, specialist and allied health services in the community by service delivery type and frequency.

The Figure 1 below offers a visual representation of the availability of primary, specialist and allied health services. In most services, high-use services requiring less specialisation tended to be the most frequently available, and low-use and highly specialised services tended to be the least frequently available.

Participants also discussed service availability. Key areas of underservicing cited by participants included poor access to cancer care, chronic healthcare, preventative oral healthcare, occupational therapy, physiotherapy and a lack of sexual health services for schools. Visiting FIFO specialists cited very advanced cases of chronic illness, indicating that there was poor access to early intervention for some patients. School staff also identified issues with early access to care.

*“He didn’t know he had diabetes… he nearly died; when he got to the doctor in [regional centre] he said ‘I don’t even know how you’re standing’… maybe need some sort of education for people who have been diagnosed cos he didn’t know anything about it”*

—School Staff, A

Local health and education providers referred to interagency collaboration, and there was evidence of services working together to improve patient access to health services. Services participated in regular interagency meetings and providers were able to refer and coordinate patient care between services.

*“We have a weekly interagency meeting where we talk about complex clients”*

—Health Service Manager, A

However, there continues to be areas where poor coordination of services and a lack of interagency cooperation resulted in underservicing and overservicing in the community. For example, one school had access to two speech pathologists, while another school reported that the lack of speech pathologists was a significant gap in healthcare access for their students. Another example was that there were two dieticians and two counsellors regularly providing services in the town; this was perceived by some participants as overservicing the needs of the community and interfering with the continuity of patient care. In another instance, a podiatrist was flown in to work specifically with Aboriginal patients with chronic illnesses, but because they were unable to extend this service to Aboriginal patients without a chronic illness and non-Aboriginal patients with a chronic illness, another health service in the community had to fly in a second podiatrist to provide the same care to these other patients. Staff cited funding structures as a significant driver for doubling up of services in the community, as services struggled to maintain staff numbers without taking every funding opportunity available.

*“There is a lack of integration of what [services] we’ve already got, no one is allowed to lead [interagency cooperation] … it’s not always in their interest to collaborate”*

—Health Service Manager, A

*“There is a bit of a doubling up… someone decides we need one of these things–but don’t worry if its already somewhere else… so everyone’s sort of struggling to maintain their numbers”*

—Health Service Manager, B

*“There’s a doubling up of counselling… we don’t communicate particularly, to know which day they’re there [another local health service] and which day they’re here. And I think it’d be really good if both those people could get together and … [organise days to come, coordinate care for shared patients]”*

—Health Service Manager, B

*“Nurses go to the [other] school at least once a week… but they don’t do that for us”*

—School Staff, B

### 3.2. Reliance on Nurses, Aboriginal Health Workers, Community Health Workers, Teachers and Administration Staff

Interviews with healthcare and education professionals working in the community revealed the indispensable role of nurses, community health workers, administration staff, and schools in meeting service gaps, networking to organise and provide services, and to follow up with patients, and provided transport, home care and school programs to support their patients’ needs. These health and education workers demonstrated extraordinary resilience, advocating for the needs of the community, and providing flexible solutions to improve the accessibility of healthcare and treatment for patients.

Health service providers acknowledged the importance of Aboriginal teachers, nurses and community health workers in building trust for Aboriginal patients and their families to express their needs. This was vital to providing culturally safe and appropriate support for Aboriginal people, as well as to improve their access to local health and education services.

*“I rely heavily on my Aboriginal education workers, because they’re the ones that put the foot in the door to build that relationship with the parents… and then those families don’t feel like they’re being judged or like they’ll be reported to [social services]”*

—School Staff, B

With a limited number of doctors available in the community, nurses and community health workers were perceived as essential to providing patient care. Nurses were highly trained and provided a range of services to meet the needs of the community and reduce the need for patients to travel for care. Clinical nurse consultants and midwives were able to provide more specialised services for patients and support visiting or telehealth specialists. While these services were highly valued by health service managers, there was a lack of federal and state support and funding for nurses to train as a clinical nurse consultant.

*“As part of their employment, [nurses] have to do dialysis training, because you can’t meet the demands of the roster… Nurses have to do the First Line Emergency Care Course, they have to do first emergency care, they’re pretty highly trained because… we have so many varied things coming through the door”*

—Health Service Manager, A

Administrative staff and community health workers worked within and outside the scope of their roles to improve the coordination of patient care, improve accessibility of care for patients and to ensure that patients were able to access appropriate services under federal initiatives including Closing the Gap and Healthy for Life. Healthy for Life, a program initially implemented by the Australian government in 2006–7, focused on improving key areas of Aboriginal health. The program evaluated the accessibility of primary health services among the study population and implemented service improvements [25]. Since being transferred to ACCHO control, Healthy for Life teams at various ACCHOs provide administrative, educational, support and health services to improve access to primary healthcare services for Aboriginal communities [26,27]. Administration staff reported poor infrastructure to facilitate the implementation of and access to Closing the Gap initiatives.

*“No one manages [Closing the Gap initiatives]; we just sign them up and do it”*

—Administration Staff

The community had two local schools, one public and one private, both of which took on a significant role improving healthcare access for students and their families. At both schools, school staff and visiting health professionals implemented health-promotion programs designed to improve health literacy in oral hygiene, nutrition and exercise. Schools in rural and remote areas are often utilised as a hub for service provision under the community-connected schools strategy [28]. One of the local schools in the study population was a public community-connected school. This school hosted visiting health services made available for students including nurses, occupational therapy, speech pathology, dieticians, and testing for sight and hearing.

Staff at the local private school took on a similar administrative role of connecting students with health services. Although this school was not connected with local or travelling health services, staff facilitated telehealth appointments and arranged for students and their families to travel for care. This was essential to improving the accessibility of telehealth services for students and their families.

*“If [Hospital in city] want to do a video conference, it’s done here at school. We sit in with them [the parents/carers], so we know what’s being said, because sometimes they use language that the parents don’t fully understand, then after the session… I explain what that actually means. And then in some cases we will implement programs at the school to help the parents”*

—School Staff, C

### 3.3. DIDO, FIFO and Telehealth

People in the community needed access to specialist services to manage chronic health conditions. DIDO, FIFO and telehealth services were considered essential for providing this specialised care and reducing the need for patients to travel for health services. While local health services were able to provide most primary health services and some specialised nurse-run services all of the time, no specialised health professionals and few allied health professionals were available locally all of the time. As seen in Table 1 and Charts 1, 2 and 3, DIDO and FIFO services made up a large proportion of healthcare and were utilised for primary, allied and specialised health services. In comparison, telehealth was utilised much less frequently and was used mostly for emergency and specialised healthcare.

*“I don’t know what the solution is, we just need to have services when they’re required”*

—School Staff, C

Staff expressed mixed levels of confidence in the appropriateness of travelling and telehealth services to meet the community needs. Some staff felt that specialist medical services were available at a comparable rate to cities, where waiting lists impact the accessibility of these services. Other staff felt that it would be better to have more of these services in the community full time with a focus on preventative care, rather than only providing complex care to patients with very advanced cases of chronic disease.

*“I think as a town we have a lot of services that come here. If you’re in the city there are some of these people [specialists] you can’t get into for months and months. I’ve seen people here wanting to see the ENT walking straight in to see the ENT. That doesn’t happen anywhere else”*

—ACCHO staff

In 2016, at the time of data collection, telehealth was starting to be utilised by health services to improve access to specialist health services, and to provide risk assessments for emergency health, emergency mental health and detox services. Service providers and health workers expressed concerns about the appropriateness of telehealth for their patients, as well as the poor coordination, lack of training and lack of consultation during the implementation of these services. Despite these concerns, the same participants acknowledged the usefulness of telehealth, particularly for follow ups with patients and for providing support in emergency health.

*“[Telehealth], it’s the way to go, they’re putting so much money into it and the Chief Executive is so into it… with mental health [risk assessment support] it’s fantastic… [but it] isn’t happening properly yet…it’s not well coordinated and they’re not doing any consultation, but the technology is there”*

—Health Service Manager, B

## 4. Discussion

### 4.1. Service Availability

A consistent theme discussed by participants was the poor coordination of services and resources between health services, and that collaboration between local health services was not facilitated. Our findings suggest this resulted in both apparent underservicing and overservicing in key areas of health provision. It is likely that this issue stemmed from fractured funding and allocation of resources for the three major local health services. As these health services received their primary funding from different sources and competed over joint funding for specific health initiatives, there was often poor coordination about which services should be available in which locations, and few incentives to cooperate or share resources.

When funding was available to bring in specialised health services, these services often utilised this without coordinating with other local services, resulting in the doubling of dieticians, counsellors, and podiatrists, where one of each could have been a more efficient use of resources and staffing. Coordinating these services would reduce travel costs and improve the continuity of patient care and service delivery for the whole community. Community interagency meetings are an existing structure through which the coordination of services could be facilitated, but this is unlikely to happen without independent leadership to manage resource allocation and funding concerns without bias, or perceived bias, towards their own health service.

### 4.2. Reliance on Nurses, Community Health Workers, Teachers and Admin Staff

The findings highlighted the significant reliance on nurses, Aboriginal health workers, community health workers, school staff and health administration staff to meet gaps in healthcare provision, coordination and patient advocacy. Much of this work could be augmented and supported through more targeted and consistent funding and support for these services, including providing opportunities for further training and succession planning.

One significant gap was the lack of specific funding and support for the management, coordination and administrative work associated with Closing the Gap initiatives. The local ACCHO staff have taken responsibility for ensuring their patients have access to the services and benefits offered under Closing the Gap. To improve the capacity of the ACCHO staff to perform this task alongside their traditional scope of practice, it would be beneficial for the State/Territory or Federal governments to fund one or more positions to facilitate service access under Closing the Gap. The capacity for this role was demonstrated with the comparable Healthy for Life program, through which two nurses in the study community were employed at the local ACCHO specifically to perform health checks and to coordinate and support patient care under the program.

The findings indicated that training and upskilling nurses and Aboriginal health workers was essential to providing healthcare to meet community needs. For example, the specialised training in dialysis for nurses was reported to have reduced the burden on patients with chronic diseases to travel for this service. This finding is consistent with previous research which has highlighted the capacity of nurse practitioners and other staff to improve the efficiency and capacity of healthcare in remote health services [29,30]. In rural and remote communities, it is typical for there to be a much higher nurse-to-medical-practitioner ratio than in cities: 6.2:1 in very remote communities in comparison to 3.2:1 in cities [31]. Therefore, upskilling nurses is vital to improving the delivery of a range of services available to patients locally. There is also a need to further develop para-professional staff to support local service delivery. Approaches such as those described by Gwynne and Lincoln and Deroy and Schütze may be useful and further research is required [29,30].

The importance of school staff to facilitate access to healthcare for their students, promote healthy behaviours and support families to meet their children’s health needs was also highlighted. While a range of health services were available to students at the local public school, the lack of interagency collaboration between the two local schools meant that not all children in the community benefitted from access to these services locally. Despite these challenges, school staff went beyond their traditional roles as educators, and acted as health advocates for their students, helping families access services, understand health information, and implementing programs to assist students in following treatment plans. It would be beneficial for further research to be conducted into the feasibility of supporting this role, potentially through providing and incentivising training in health literacy and health advocacy for teachers in rural and remote schools.

### 4.3. DIDO, FIFO and Telehealth

DIDO, FIFO, and telehealth services are an important component of providing specialised health services in rural and remote communities. These services reduce the need for patients to travel to access care, which has is a significant barrier to healthcare access, particularly for Aboriginal people living in rural and remote communities [21]. For travelling specialist services to adequately meet the needs of the community, these services need to be linked to local and adequate primary and allied health services to provide primary care and education for patients and support ongoing patient treatment [32,33].

For example, the service availability tables demonstrated that mental health is supported by access to a local mental health trainee, counsellors who drive in weekly, and specialised mental health services including a psychologist, a psychiatrist and a psychoneurologist who attend the community less frequently. This decreasing frequency of availability with increasing expertise improves the utilisation of these resources, ensuring a strong foundation of primary and allied health services available to meet the needs of most patients. This ensures specialists have time to focus on complex cases where their expertise is invaluable.

The participants of this study had polarised views on FIFO health services. This is reflective of the varying capacity of FIFO services to meet the health needs of rural and remote communities. Previous research has highlighted how travelling health services can be optimised by ensuring that services are targeted towards supporting the health needs of the receiving community and strengthening local healthcare systems [32,33].

While travelling health services are vital for meeting patient needs in rural and remote communities, these services are expensive to run and are not always able to provide an adequate continuity of care for patients [33]. They are also not appropriate for sporadically needed services, particularly emergency medicine. This has led to the growing utilisation of telehealth services to meet these needs [34].

In 2016, at the time of the data collection, the use of telehealth services was just emerging. Health professionals could see several benefits of telehealth, particularly for follow-ups with specialists and risk assessment for emergency care. However, they also expressed concerns about the poor consultation of local health professionals and patients, and a lack of training to facilitate the use of telehealth systems.

The capacity and use of telehealth have increased significantly in recent years, particularly during COVID-19, when telehealth was used as the primary means for outpatient care [35]. As telehealth continues to develop, it is likely to become an even more important part of rural and remote medicine. Research has demonstrated the many benefits telehealth has for Aboriginal patients, by improving access to more flexible and high-quality healthcare, improving clinical outcomes and reducing costs [36].

Similarly, COVID-19 is likely to have impacted the availability of FIFO and DIDO health services. Further research is needed to understand changes in the availability, accessibility and delivery of key health services in remote locations, and their impact on the health of Aboriginal and Torres Strait Islander people [37].

### 4.4. Strengths and Limitations

A strength of this study is the use of the co-design method, which ensured that the research was relevant to the community and reflected their key concerns. Another strength was the broad inclusion of participants in this study, who performed different roles in the health system. This provided a more holistic picture of the health system in the community.

A limitation of the study was the small sample size, 11 participants, which focused on data from only one remote community in NSW and therefore cannot be fully representative of remote health in Australia. Additionally, as the data were collected in 2016, the study provides a snapshot of service availability at this time, and this may have changed since then. One key area of significant change since the data were collected is the increased utilisation of telehealth, particularly in the context of COVID-19. We have mediated this limitation by discussing the implications of our findings in the context of the changing role of telehealth.

## 5. Conclusions

This study has focused on the health system of a remote town in Australia, mapping access to 40 health services, describing their varying levels of availability through measures of frequency and locality. Interviews with health and education professionals revealed three key themes about rural and remote healthcare. Analysis of the health services available to the community demonstrated key issues in overservicing and underservicing. Poor incentivisation and resourcing of service coordination between health providers was cited as a key barrier to interagency cooperation. Local health and education staff, particularly nurses, Aboriginal health workers, school staff, administrative staff, and community health workers, showed extraordinary resilience and commitment to making health services accessible to the community. Visiting and telehealth services provided access to specialised and allied healthcare services, which were most effective when local services had the training, resources, and capacity to provide ongoing support and continuity of patient care.

## Figures and Tables

**Table 1:** Availability of services by location.

	Local	DIDO	FIFO	Telehealth	Patients Required to Travel
Aboriginal Educator					
Aboriginal Health Worker					
Aboriginal Nurse					
Addiction Medicine					
Audiology					
Cardiology					
Chiropody					
Clinical Nurse Specialist					
Community Health					
Counselling					
Dentistry					
Diabetes Educator					
Diabetes Medicine					
Dietician/Nutritionist					
Ear, Nose and Throat					
Endocrinology					
Family Health					
General Practitioner					
Geriatric Medicine					
Mental Health Trainee					
Midwifery					†
Neurology					
Occupational Therapy					
Optometry					‡
Oral Hygienist					
Paediatric Medicine					
Pharmacist					
Physical Trainer					
Physiotherapy					
Psychiatry					
Psychology					
Psychoneurology					
Registered Nurse					
Renal Medicine		§			
Respiratory Medicine					
Sexual Health					
Social Worker					
Sonography					¶
Speech Pathology					
Sports Medicine/Exercise Physiologist					

† Patient required to travel for birth of child. ‡ Patient required to travel for laser surgery. § Available monthly, may be either FIFO or DIDO. ¶ Patient required to travel for ultrasounds.

**Table 2:** Frequency of service availability locally.

	Always	Weekly	Fortnightly	Monthly	Quarterly	Yearly
Aboriginal Educator						
Aboriginal Health Worker						
Aboriginal Nurse						
Addiction Medicine						
Audiology						
Cardiology				†		
Chiropody						
Clinical Nurse Specialist						
Community Health						
Counselling						
Dentistry				‡		
Diabetes Educator						
Diabetes Medicine						
Dietician/Nutritionist						
Ear, Nose and Throat						
Endocrinology						
Family Health						
General Practitioner						
Geriatric Medicine						
Mental Health Trainee						
Midwifery						
Neurology						§
Occupational Therapy						
Optometry				¶	††	
Oral Hygienist						
Paediatric Medicine						
Pharmacist						
Physical Trainer						
Physiotherapy						
Psychiatry						
Psychology						
Psychoneurology						
Registered Nurse						
Renal Medicine						
Respiratory Medicine						
Sexual Health				†‡		
Social Worker						
Sonography						
Speech Pathology						
Sports Medicine/ Exercise Physiologist						

† Every 6 weeks. ‡ Student dentists provide care to adult patients only, and come in 6-week blocks, 4–5 times per year. § Twice per year. ¶ Every 3 weeks. †† Ophthalmologist. †‡ Every two months.

## Data Availability

Data are available upon request to the authors and conditional on ethics approval.
